# 1,3-Bis(2-nitro­phen­oxy)propan-2-ol

**DOI:** 10.1107/S1600536811000250

**Published:** 2011-01-12

**Authors:** Zhi-You Xiao, Wen-Hua Tang, Shao-Yun Wang

**Affiliations:** a School of Environment and Life Sciences, Kaili University, Kaili, Guizhou 556000, People’s Republic of China

## Abstract

In the title compound, C_15_H_14_N_2_O_7_, the planes of the two benzene rings form a dihedral angle of 33.16 (17)°. In the crystal, inter­molecular hydrogen bonds involveing the OH group and nitro O atoms link the mol­ecules into chains  propagating along the *a* axis.

## Related literature

For a related structure, see: Elizondo *et al.* (2009 ▶). For general background to the use of amines as inter­mediates in the preparation of dyes, herbicides, pesticides, and pharmaceuticals, see: Downing *et al.* (1997 ▶); Tafesh *et al.* (1996 ▶).
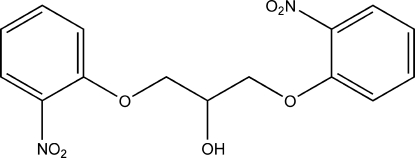


## Experimental

### 

#### Crystal data

C_15_H_14_N_2_O_7_
*M* *_r_* = 334.28
Tetragonal, 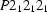

*a* = 7.287 (4) Å
*c* = 28.158 (17) Å
*V* = 1495.2 (13) Å^3^
*Z* = 4
Mo *K*α radiation
μ = 0.12 mm^−1^
*T* = 293 K
0.22 × 0.19 × 0.18 mm

#### Data collection

Bruker SMART CCD area-detector diffractometer
Absorption correction: multi-scan (*SADABS*; Bruker, 2005 ▶) *T*_min_ = 0.974, *T*_max_ = 0.979
10200 measured reflections
1570 independent reflections
1022 reflections with *I* > 2σ(*I*)
*R*_int_ = 0.062

#### Refinement

*R*[*F* ^2^ > 2σ(*F* ^2^)] = 0.039
*wR*(*F* ^2^) = 0.103
*S* = 0.96
1570 reflections
218 parameters
H-atom parameters constrained
Δρ_max_ = 0.15 e Å^−3^
Δρ_min_ = −0.15 e Å^−3^

### 

Data collection: *SMART* (Bruker, 2002 ▶); cell refinement: *SAINT* (Bruker, 2002 ▶); data reduction: *SAINT*; program(s) used to solve structure: *SHELXS97* (Sheldrick, 2008 ▶); program(s) used to refine structure: *SHELXL97* (Sheldrick, 2008 ▶); molecular graphics: *ORTEP-3 for Windows* (Farrugia, 1997 ▶); software used to prepare material for publication: *WinGX* (Farrugia, 1999 ▶).

## Supplementary Material

Crystal structure: contains datablocks global, I. DOI: 10.1107/S1600536811000250/bq2262sup1.cif

Structure factors: contains datablocks I. DOI: 10.1107/S1600536811000250/bq2262Isup2.hkl

Additional supplementary materials:  crystallographic information; 3D view; checkCIF report

## Figures and Tables

**Table 1:** Hydrogen-bond geometry (Å, °)

*D*—H⋯*A*	*D*—H	H⋯*A*	*D*⋯*A*	*D*—H⋯*A*
O4—H4*A*⋯O6^i^	0.82	2.43	3.079 (4)	137

Symmetry code: (i) 
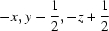
.

**Fig. 1.**
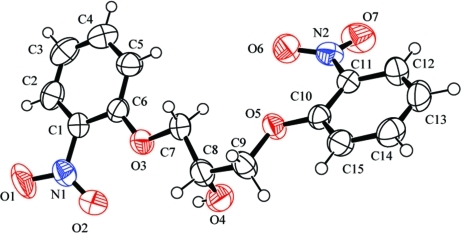
The molecular structure of the title compound showing the atom-labeling scheme. Displacement ellipsoids are drawn at the 50% probability level.

**Fig. 2.**
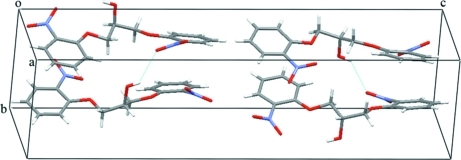
Crystal packing viewed down the a axis.

**Fig. 3.**
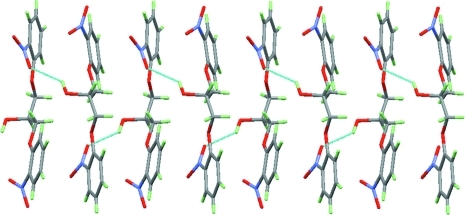
Intermolecular hydrogen link the molecules into a chains propagating along a axis. Hydrogen bonds are shown with dashed lines.

## supplementary crystallographic information

### Comment

Aromatic nitro compounds can be reduced to the corresponding amines, which are important intermediates in the preparation of dyes, herbicides, pesticides, and pharmaceuticals (Tafesh *et al.*, 1996; Downing *et al.*, 1997; Elizondo *et al.* 2009). Herein we report the crystal structure of the title compound. The crystal structure of the title compound is represented in Fig. 1. The aromatic rings in a molecule are not coplanar, and the planes of two benzene rings form dihedral angle of 33.16 (17) °. the nitro groups are twisted out of the planes of their attached benzene rings, and the dihedral angles are 30.8 (2) and 24.25 (19), respectively. The O—H···O hydrogen bonds are observed between OH-group and the nitro groups O atom, the distance of the O4—H4A···O6 hydrogen bonds is 3.079 (4) Å(Table 1). These intermolecular H-bond links the molecules into supramolecular structure, and crystal packing of the title compound is shown in Fig. 2.

### Experimental

Epichlorohydrin (2.31 g, 0.025 mol) was added slowly to a stirred solution of NaOH (2.0 g,0.05 mol), *O*-nitrophenol (6.95 g,0.05 mol) in water (40 ml) at 60°C over a period of 40 min, and the solution was stirred at 60°C for 3 h, then the solvent was removed by reduced pressure filter, the solid product was rinsed with 30 ml water, then the crude product was dissolved in 50 ml 95% ethanol solution, and then set aside for five days to obtain 3.1 g light yellow crystals, in a yield of 36%.

### Refinement

H atoms were placed in calculated positions and refined as riding, with C—H = 0.93–0.98 Å, O—H = 0.82 Å, and and *U*_iso_(H) = 1.2*U*_eq_(C) or 1.5*U*_eq_(O).

### Crystal data

**Table:**

C_15_H_14_N_2_O_7_	*D*_x_ = 1.485 Mg m^−^^3^
*M**_r_* = 334.28	Mo *K*α radiation, λ = 0.71073 Å
Tetragonal, *P*2_1_2_1_2_1_	Cell parameters from 1570 reflections
Hall symbol: P 2ac 2ab	θ = 1.5–25.0°
*a* = 7.287 (4) Å	µ = 0.12 mm^−^^1^
*c* = 28.158 (17) Å	*T* = 293 K
*V* = 1495.2 (13) Å^3^	Block, yellow
*Z* = 4	0.22 × 0.19 × 0.18 mm
*F*(000) = 696	

### Data collection

**Table:**

Bruker SMART CCD area-detector diffractometer	1570 independent reflections
Radiation source: fine-focus sealed tube	1022 reflections with *I* > 2σ(*I*)
graphite	*R*_int_ = 0.062
φ and ω scans	θ_max_ = 25.0°, θ_min_ = 1.5°
Absorption correction: multi-scan (*SADABS*; Bruker, 2005)	*h* = −7→8
*T*_min_ = 0.974, *T*_max_ = 0.979	*k* = −8→8
10200 measured reflections	*l* = −33→32

### Refinement

**Table:**

Refinement on *F*^2^	Secondary atom site location: difference Fourier map
Least-squares matrix: full	Hydrogen site location: inferred from neighbouring sites
*R*[*F*^2^ > 2σ(*F*^2^)] = 0.039	H-atom parameters constrained
*wR*(*F*^2^) = 0.103	*w* = 1/[σ^2^(*F*_o_^2^) + (0.0581*P*)^2^] where *P* = (*F*_o_^2^ + 2*F*_c_^2^)/3
*S* = 0.96	(Δ/σ)_max_ < 0.001
1570 reflections	Δρ_max_ = 0.15 e Å^−^^3^
218 parameters	Δρ_min_ = −0.15 e Å^−^^3^
0 restraints	Extinction correction: *SHELXL97* (Sheldrick, 2008), Fc^*^=kFc[1+0.001xFc^2^λ^3^/sin(2θ)]^-1/4^
Primary atom site location: structure-invariant direct methods	Extinction coefficient: 0.0123 (17)

### Special details

**Table:**

Geometry. All e.s.d.'s (except the e.s.d. in the dihedral angle between two l.s. planes) are estimated using the full covariance matrix. The cell e.s.d.'s are taken into account individually in the estimation of e.s.d.'s in distances, angles and torsion angles; correlations between e.s.d.'s in cell parameters are only used when they are defined by crystal symmetry. An approximate (isotropic) treatment of cell e.s.d.'s is used for estimating e.s.d.'s involving l.s. planes.
Refinement. Refinement of *F*^2^ against ALL reflections. The weighted *R*-factor *wR* and goodness of fit *S* are based on *F*^2^, conventional *R*-factors *R* are based on *F*, with *F* set to zero for negative *F*^2^. The threshold expression of *F*^2^ > σ(*F*^2^) is used only for calculating *R*-factors(gt) *etc*. and is not relevant to the choice of reflections for refinement. *R*-factors based on *F*^2^ are statistically about twice as large as those based on *F*, and *R*- factors based on ALL data will be even larger.

### Fractional atomic coordinates and isotropic or equivalent isotropic displacement parameters (Å^2^)

**Table:**

	*x*	*y*	*z*	*U*_iso_*/*U*_eq_	
C1	0.0232 (5)	0.8105 (5)	0.40291 (10)	0.0467 (9)	
C2	−0.1155 (6)	0.8419 (5)	0.43548 (12)	0.0594 (10)	
H2	−0.0936	0.8266	0.4678	0.071*	
C3	−0.2854 (6)	0.8957 (5)	0.41980 (14)	0.0709 (12)	
H3	−0.3794	0.9164	0.4415	0.085*	
C4	−0.3162 (6)	0.9188 (6)	0.37265 (14)	0.0698 (11)	
H4	−0.4329	0.9519	0.3623	0.084*	
C5	−0.1785 (5)	0.8944 (5)	0.33975 (12)	0.0589 (10)	
H5	−0.2014	0.9155	0.3077	0.071*	
C6	−0.0061 (5)	0.8383 (5)	0.35432 (10)	0.0476 (9)	
C7	0.1259 (5)	0.8721 (5)	0.27648 (9)	0.0539 (10)	
H7A	0.1111	1.0041	0.2742	0.065*	
H7B	0.0226	0.8130	0.2610	0.065*	
C8	0.3040 (5)	0.8121 (5)	0.25460 (11)	0.0552 (9)	
H8	0.4052	0.8645	0.2731	0.066*	
C9	0.3240 (4)	0.8721 (5)	0.20369 (10)	0.0561 (10)	
H9A	0.3520	1.0021	0.2023	0.067*	
H9B	0.4230	0.8051	0.1885	0.067*	
C10	0.1474 (5)	0.8550 (5)	0.13208 (10)	0.0433 (8)	
C11	−0.0153 (4)	0.8131 (5)	0.10830 (10)	0.0427 (8)	
C12	−0.0277 (5)	0.8204 (5)	0.05975 (11)	0.0576 (10)	
H12	−0.1371	0.7895	0.0447	0.069*	
C13	0.1211 (6)	0.8731 (5)	0.03335 (11)	0.0629 (11)	
H13	0.1133	0.8780	0.0004	0.076*	
C14	0.2825 (6)	0.9190 (5)	0.05595 (11)	0.0576 (10)	
H14	0.3828	0.9573	0.0381	0.069*	
C15	0.2969 (5)	0.9087 (4)	0.10472 (11)	0.0504 (9)	
H15	0.4074	0.9379	0.1194	0.060*	
N1	0.2012 (4)	0.7496 (4)	0.42086 (10)	0.0571 (8)	
N2	−0.1829 (4)	0.7619 (4)	0.13400 (11)	0.0553 (8)	
O1	0.2440 (4)	0.7981 (4)	0.46074 (9)	0.0978 (11)	
O2	0.2959 (4)	0.6490 (4)	0.39666 (9)	0.0702 (8)	
O3	0.1401 (3)	0.8157 (4)	0.32490 (7)	0.0598 (7)	
O4	0.3173 (4)	0.6194 (3)	0.25628 (7)	0.0711 (8)	
H4A	0.3071	0.5847	0.2839	0.107*	
O5	0.1533 (3)	0.8355 (3)	0.17982 (7)	0.0510 (6)	
O6	−0.2026 (3)	0.8110 (4)	0.17517 (8)	0.0701 (8)	
O7	−0.2971 (4)	0.6716 (4)	0.11246 (10)	0.0813 (8)	

### Atomic displacement parameters (Å^2^)

**Table:**

	*U*^11^	*U*^22^	*U*^33^	*U*^12^	*U*^13^	*U*^23^
C1	0.053 (2)	0.045 (2)	0.0417 (18)	−0.0052 (19)	0.0001 (17)	−0.0017 (15)
C2	0.072 (3)	0.056 (2)	0.050 (2)	−0.007 (2)	0.014 (2)	−0.0026 (19)
C3	0.068 (3)	0.065 (3)	0.080 (3)	0.000 (3)	0.030 (2)	−0.005 (2)
C4	0.059 (3)	0.069 (3)	0.081 (3)	0.011 (2)	0.008 (2)	0.003 (2)
C5	0.058 (3)	0.062 (2)	0.057 (2)	0.009 (2)	0.000 (2)	0.0034 (18)
C6	0.047 (2)	0.050 (2)	0.0457 (19)	−0.0015 (19)	0.0053 (17)	−0.0067 (16)
C7	0.061 (2)	0.065 (2)	0.0355 (17)	0.006 (2)	−0.0027 (16)	−0.0052 (16)
C8	0.050 (2)	0.072 (3)	0.0440 (18)	−0.005 (2)	−0.0023 (18)	−0.0010 (18)
C9	0.042 (2)	0.082 (3)	0.0443 (19)	−0.010 (2)	−0.0042 (16)	0.0027 (18)
C10	0.047 (2)	0.047 (2)	0.0362 (17)	0.0020 (19)	0.0045 (16)	0.0021 (14)
C11	0.042 (2)	0.045 (2)	0.0414 (17)	0.0013 (17)	0.0007 (16)	−0.0012 (16)
C12	0.065 (2)	0.063 (3)	0.0451 (19)	0.003 (2)	−0.0075 (19)	−0.0072 (18)
C13	0.084 (3)	0.067 (3)	0.0380 (19)	0.012 (3)	0.003 (2)	0.0003 (18)
C14	0.059 (3)	0.063 (2)	0.050 (2)	0.004 (2)	0.017 (2)	0.0098 (18)
C15	0.046 (2)	0.059 (2)	0.0457 (18)	0.000 (2)	0.0048 (17)	0.0058 (17)
N1	0.058 (2)	0.069 (2)	0.0447 (17)	−0.0081 (19)	−0.0046 (16)	0.0048 (15)
N2	0.0434 (19)	0.061 (2)	0.0613 (19)	0.0023 (18)	−0.0029 (17)	0.0045 (16)
O1	0.104 (2)	0.144 (3)	0.0458 (15)	−0.002 (2)	−0.0294 (15)	−0.0091 (16)
O2	0.0593 (18)	0.0792 (19)	0.0721 (16)	0.0094 (18)	−0.0089 (14)	−0.0002 (15)
O3	0.0565 (15)	0.0866 (18)	0.0363 (12)	0.0130 (15)	0.0000 (11)	0.0020 (12)
O4	0.0822 (19)	0.0783 (19)	0.0527 (14)	0.0236 (17)	0.0045 (13)	0.0071 (12)
O5	0.0410 (13)	0.0744 (16)	0.0377 (12)	−0.0050 (13)	−0.0011 (10)	0.0034 (11)
O6	0.0509 (16)	0.105 (2)	0.0544 (14)	−0.0034 (17)	0.0118 (13)	0.0020 (15)
O7	0.0492 (17)	0.089 (2)	0.105 (2)	−0.0169 (18)	−0.0067 (16)	−0.0063 (18)

### Geometric parameters (Å, °)

**Table:**

C1—C2	1.383 (4)	C9—H9A	0.9700
C1—C6	1.399 (4)	C9—H9B	0.9700
C1—N1	1.461 (4)	C10—O5	1.352 (3)
C2—C3	1.372 (5)	C10—C15	1.390 (4)
C2—H2	0.9300	C10—C11	1.396 (4)
C3—C4	1.357 (5)	C11—C12	1.371 (4)
C3—H3	0.9300	C11—N2	1.468 (4)
C4—C5	1.378 (5)	C12—C13	1.370 (5)
C4—H4	0.9300	C12—H12	0.9300
C5—C6	1.383 (5)	C13—C14	1.378 (5)
C5—H5	0.9300	C13—H13	0.9300
C6—O3	1.359 (4)	C14—C15	1.379 (4)
C7—O3	1.428 (3)	C14—H14	0.9300
C7—C8	1.502 (4)	C15—H15	0.9300
C7—H7A	0.9700	N1—O2	1.216 (3)
C7—H7B	0.9700	N1—O1	1.218 (3)
C8—O4	1.408 (4)	N2—O6	1.222 (3)
C8—C9	1.506 (4)	N2—O7	1.222 (3)
C8—H8	0.9800	O4—H4A	0.8200
C9—O5	1.439 (3)		
			
C2—C1—C6	120.9 (3)	C8—C9—H9A	110.1
C2—C1—N1	118.0 (3)	O5—C9—H9B	110.1
C6—C1—N1	121.2 (3)	C8—C9—H9B	110.1
C3—C2—C1	119.5 (3)	H9A—C9—H9B	108.4
C3—C2—H2	120.2	O5—C10—C15	123.8 (3)
C1—C2—H2	120.2	O5—C10—C11	118.7 (3)
C4—C3—C2	120.0 (4)	C15—C10—C11	117.5 (3)
C4—C3—H3	120.0	C12—C11—C10	121.7 (3)
C2—C3—H3	120.0	C12—C11—N2	116.5 (3)
C3—C4—C5	121.4 (4)	C10—C11—N2	121.8 (3)
C3—C4—H4	119.3	C13—C12—C11	120.0 (3)
C5—C4—H4	119.3	C13—C12—H12	120.0
C4—C5—C6	120.0 (3)	C11—C12—H12	120.0
C4—C5—H5	120.0	C12—C13—C14	119.5 (3)
C6—C5—H5	120.0	C12—C13—H13	120.2
O3—C6—C5	124.5 (3)	C14—C13—H13	120.2
O3—C6—C1	117.3 (3)	C13—C14—C15	120.7 (3)
C5—C6—C1	118.1 (3)	C13—C14—H14	119.6
O3—C7—C8	104.2 (3)	C15—C14—H14	119.6
O3—C7—H7A	110.9	C14—C15—C10	120.5 (3)
C8—C7—H7A	110.9	C14—C15—H15	119.7
O3—C7—H7B	110.9	C10—C15—H15	119.7
C8—C7—H7B	110.9	O2—N1—O1	123.1 (3)
H7A—C7—H7B	108.9	O2—N1—C1	119.6 (3)
O4—C8—C7	109.6 (3)	O1—N1—C1	117.3 (3)
O4—C8—C9	108.4 (3)	O6—N2—O7	123.3 (3)
C7—C8—C9	112.9 (3)	O6—N2—C11	119.4 (3)
O4—C8—H8	108.6	O7—N2—C11	117.3 (3)
C7—C8—H8	108.6	C6—O3—C7	119.3 (3)
C9—C8—H8	108.6	C8—O4—H4A	109.5
O5—C9—C8	107.9 (3)	C10—O5—C9	118.2 (2)
O5—C9—H9A	110.1		

### Hydrogen-bond geometry (Å, °)

**Table:**

*D*—H···*A*	*D*—H	H···*A*	*D*···*A*	*D*—H···*A*
O4—H4A···O6^i^	0.82	2.43	3.079 (4)	137.

Symmetry codes: (i) −*x*, *y*−1/2, −*z*+1/2.
